# ‘Desperate house genes’: the dramatic example of hypoxia

**DOI:** 10.1038/sj.bjc.6605573

**Published:** 2010-02-23

**Authors:** J Caradec, N Sirab, C Keumeugni, S Moutereau, M Chimingqi, C Matar, D Revaud, M Bah, P Manivet, M Conti, S Loric

**Affiliations:** 1 INSERM, U955 EQ07, Créteil, France; 2 Paris Est University, Créteil, France; 3 Bioquanta Corp, Aurora, CO, USA; 4 Clinical Biochemistry and Genetics Department, Mondor University Hospital, Créteil, France; 5 Clinical Chemistry Laboratory, Lariboisière University Hospital, Paris, France; 6 INSERM UEVE 829, Evry, France; 7 Clinical Chemistry Laboratory, Bicêtre University Hospital, Le Kremlin Bicêtre, France

**Keywords:** housekeeping genes, normalisation, RNA, PCR, quantification

## Abstract

**Background:**

Microenvironmental conditions in normal or tumour tissues and cell lines may interfere on further biological analysis. To evaluate transcript variations carefully, it is common to use stable housekeeping genes (HKG) to normalise quantitative microarrays or real-time polymerase chain reaction results. However, recent studies argue that HKG fluctuate according to tissues and treatments. So, as an example of HKG variation under an array of conditions that are common in the cancer field, we evaluate whether hypoxia could have an impact on HKG expression.

**Methods:**

Expression of 10 commonly used HKG was measured on four cell lines treated with four oxygen concentrations (from 1 to 20%).

**Results:**

Large variations of HKG transcripts were observed in hypoxic conditions and differ along with the cell line and the oxygen concentration. To elect the most stable HKG, we compared the three statistical means based either on PCR cycle threshold coefficient of variation calculation or two specifically dedicated software. Nevertheless, the best HKG dramatically differs according to the statistical method used. Moreover, using, as a reference, absolute quantification of a target gene (here the proteinase activating receptor gene 1 (*PAR1*) gene), we show that the conclusions raised about *PAR1* variation in hypoxia can totally diverge according to the selected HKG used for normalisation.

**Conclusion:**

The choice of a valid HKG will determine the relevance of the results that will be further interpreted, and so it should be seriously considered. The results of our study confirm unambiguously that HKG variations must be precisely and systematically determined before any experiment for each situation, to obtain reliable normalised results in the experimental setting that has been designed. Indeed, such assay design, functional for all *in vitro* systems, should be carefully evaluated before any extension to other experimental models including *in vivo* ones.

Gene expression analysis is becoming increasingly important as understanding gene expression patterns is expected to reveal complex regulatory networks involved in disease initiation or progression. Nowadays, two main techniques are used to evaluate gene expression: (1) microarray analysis, which allows qualitative parallel comparative analysis of thousands of target genes in two specific sets of RNA, (2) reverse-transcription (RT-PCR), which allows qualitative or semi-quantitative amplification analysis of limited sets of expressed genes in many different RNA samples. However, despite the greater interest raised by these techniques in the medical community, results may often be interpreted with caution as many conflicting reports regarding the same gene or set of gene expression have been published (Dheda *et al*, 2005; Strauss, 2006). One of their most important drawbacks is the lack of standardisation as no quality controls like the ones daily used routinely in clinical chemistry laboratories do exist (Eisenstein, 2006). Indeed, amount of starting material, RNA extraction threshold, enzyme efficiencies at the time of RT or PCR and differences in transcriptional activities of tissues or cell lines largely account for the final result but may undergo in-house variations that must be measured on a standardised basis (Nolan *et al*, 2006). Thus, standardisation protocols are mandatory (Brazma *et al*, 2001; Bustin *et al*, 2009).

One way to standardise is to report every gene expression to the extracted total RNA mass. However, as total RNA mass principally consisted in ribosomal RNA (rRNA) that rarely reflects messenger RNA (mRNA) amount, neither 18S nor 28S rRNA molecules are still used to standardise mRNA expression (Spanakis and Brouty-Boye, 1994). To date, internal control genes – also named housekeeping genes (HKG) whose expression is assumed to be and stay constant between cells under different experimental conditions – are frequently used to monitor mRNA amount. Most of HKG are chosen among cellular maintenance genes that are ubiquitously expressed and whose expression is generally considered as constant. Nevertheless, some authors have reported that HKG expression could rise and/or fall (Thellin *et al*, 1999). For example, two widely used HKG, namely glyceraldehyde-3-phosphate dehydrogenase (*GAPDH*) and *β*-actin (*ACTB*), show great *in vitro* variations (Schmittgen and Zakrajsek, 2000; Lupberger *et al*, 2002; Aerts *et al*, 2004; Dheda *et al*, 2004; Synnergren *et al*, 2007). This holds also true *in vivo* as shown in human tissues such as kidney (Schmid *et al*, 2003; Biederman *et al*, 2004), melanoma (Giricz *et al*, 2008) or hepatocellular carcinoma (Cicinnati *et al*, 2008). In addition, reporting the uneven expression of 13 HKG in a panel of normal and tumour human tissues, de Kok *et al* (2005) have shown that the less variable HKG differed according to the tissue and suggested that it will be important, before experiments, to establish HKG variations clearly to select the best one for quantitative real-time PCR (qRT-PCR) standardisation.

Many publications dealing with cancer have reported gene expression studies in hypoxic conditions (Helczynska *et al*, 2008; Seifeddine *et al*, 2008; Sheikh *et al*, 2008; Chaudary and Hill, 2009; Zhang *et al*, 2009), but until now related HKG variations have not yet been much characterised (Zhong and Simons, 1999). However, analysing if HKG expression is stable under different oxygen concentrations remains an important issue especially in cancer field as it is well known that proliferating tumours often grow in hypoxic conditions (Semenza, 2003). Thus, the aim of this study was to examine HKG variations in cell lines of different origins, cultured under different oxygen concentrations. The expression pattern of 10 commonly used HKG (namely ATP synthase (*ATP5G3*), *β*-2-microglobulin (*B2M*), *ACTB*, *β*-glucuronidase (GUSB), cyclophilin A (*PPIA*), *GAPDH*, hypoxanthine ribosyltransferase (*HPRT1*), phosphoglycerokinase (*PGK1*), TATA-box-binding protein (*TBP*) and transferrin receptor (*TFRC*)) was investigated by qRT-PCR in different cell lines cultured in various hypoxic or aerobic conditions.

## Materials and Methods

### Cell culture

Human prostate LNCaP and PNT2 cell lines, as well as breast cancer MCF-7 and kidney HEK293 cell lines, were used for experiments and were originally obtained from the ATCC (Rockville, MD, USA). All cell lines were cultured in RPMI 1640 except HEK, which was cultured in DMEM. Cells were supplemented with 10% FBS (Sigma, St Louis, MO, USA), under different oxygen concentrations (1, 5, 10 and 20%) during 72 h (Sanyo, Osaka, Japan). All oxygen conditions for each cell line were performed independently in triplicate.

### Total RNA isolation and reverse transcription

Total RNA from cultured cell lines was extracted using the cesium-chloride method. Briefly, cultured cells were homogenised in a 4 M guanidium thiocyanate/25 mM sodium citrate/0.5% sarcosyl solution. The homogenate was then ultracentrifuged (17 h, 35 000 r.p.m.) in a 5.7 mM cesium chloride/5 mM EDTA gradient. After centrifugation (15 000 r.p.m., 5 min) in a chloroform/isoamyl alcohol (24 : 1) solution, supernatant was collected then precipitated by centrifugation (15 000 r.p.m., 15 min) in 3 M sodium acetate (pH 5.2)/100% ethanol solution. Total RNA purity was controlled by Agilent Bioanalyzer (Massy, France). RT was systematically performed using 500 ng total RNA and universal primers at 72°C for 10 min, and then 1 h at 42°C with MMLv as reverse transcriptase (Invitrogen, Eragny, France). cDNA quality and concentration were controlled by RiboGreen fluorometric measurements (Invitrogen).

### Primer design for housekeeping genes

A total of 10 HKG (Table 1), used in many studies, were selected for gene expression analysis. All genes are constitutively expressed, most of them lacking canonical TATA box. All show independent functions in cellular maintenance, and the regulation of their expression is assumed not to be directly related. Among those studied, only *GAPDH* and *PGK1* share an identical cellular biochemical process, namely glycolysis. Specific primers (Eurofins, Edersberg, Germany) were designed by Primer3 software and validated *in silico* by Blast and Blat analysis. Each designed couple of primers was then tested *in vitro* onto human cDNAs by conventional PCR. Specificity of amplified products was checked by direct sequencing on 3130xl Genetic Analyzer (Applied Biosystems, Les Ulis, France). Designed and tested as above, forward 5′-TCAATGAAACCCTGCTCGAA-3′ and reverse 5′-GTTATTCAGGTTACTAGAGC-3′ primers were used to amplify the proteinase activating receptor gene 1 (*PAR1*).

### Quantitative real-time PCR

To measure HKG expression level, we performed qRT-PCR using the 7900 ABI system (Applied Biosystems). For experiment, 96-well PCR plates, divided into 12 columns of eight identical wells, were used. A total of 10 columns contained primers and probes for the detection of 10 HKG in duplicates. Six wells were systematically used for inter and intra-assays controls (500 and 50 ng of the same cDNA with identical set of primers). HKG amplification was detected by SYBR Green fluorescence using qRT-PCR mix containing 10 *μ*M of each primer, qPCR buffer (Applied Biosystems), and 500 ng cDNA in a 20 *μ*l total reaction volume. Amplification was performed as follow: 95°C (10 min) for primer elongation, 40 cycles of amplification at 95°C (30 s) for denaturation and 60°C (60 s) for annealing and extension. The number of PCR cycles to reach the fluorescence threshold in each sample was defined as the cycle threshold (*C*_t_). *C*_t_ values are proportional to the negative logarithm of the initial amount of input cDNA. *C*_t_ values of 10 HKG in each cell line were directly related. In addition, PAR1 mRNA expression was established on LNCaP cell line using a dedicated standard curve based on serial dilutions (10-fold) of known concentrations of PAR1 full-length transcript cloned into pCR2.1 plasmid (10^9^ to one copy per *μ*l) (Invitrogen).

All experiments were performed three times, and although *C*_t_ values did not fluctuate, the *C*_t_ mean of those three experiments was used for statistical analysis.

### Immunohistochemistry experiments

Immunohistochemistry was performed on LNCaP cells cultured as already described for 72 h in aerobic (20%) and hypoxic (1%) conditions. After PBS washes, cells were scrapped, gently cytocentrifuged (Hettich, Kirchlengern, Germany) and slides were fixed with 10 min cold acetone treatment. After 10 min with blocking buffer (Pierce), slides were incubated 1 h with anti-PAR1 primary antibody (ATAP2; Santa Cruz, Heidelberg, Germany) then for 1 h with biotinylated secondary horse anti-mouse IgG (Vector Laboratories, Burlingame, CA, USA). Between incubation steps, slides were washed in PBS/Tween 0.1%. 3-3-Diaminobenzidine (DAB SK-4100; Vector Laboratories) was used as detection system. Slides were counterstained with Harris haematoxylin.

### Statistical analysis

Statistical analyses were performed with both GraphPad Prism4 (GraphPad, Palo Alto, CA, USA) and SPSS10 (SPSS, Chicago, IL, USA) statistical software. Because most of the variables did not comply with normal Gaussian distribution as determined by Kolmogorov–Smirnov test, nonparametric Friedman test was applied to compare dependant samples of continuous variables. All *P*-values were based on two-tailed tests and the threshold to accept statistical significance was set at *α* level 0.05.

GeNorm and NormFinder, software specifically designed for normalisation, were used according to instructions. These are application tools for Microsoft Excel and are available online (NormFinder: http://www.mdl.dk/publicationsnormfinder.htm; GeNorm: http://medgen.ugent.be/~jvdesomp/genorm/).

## Results

Four phenotypically cell lines (PNT2, LNCaP, MCF-7 and HEK) were cultured under four oxygen concentrations (1, 5, 10% and aerobic). Total RNA of these cell lines was extracted and retrotranscribed into cDNA. For comparative purposes, 500 ng of total RNA and cDNA was systematically used for experiments. Ten HKG (Table 1) were then quantified by qRT-PCR.

### HKG variability based on PCR cycle threshold is induced by hypoxic conditions

**Figure 1:**
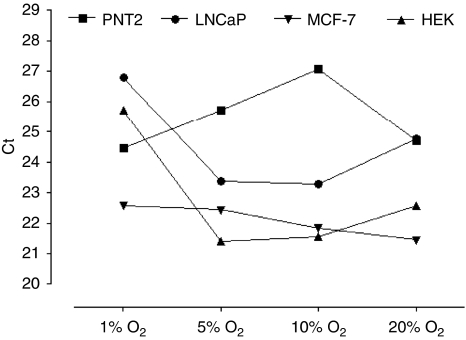
Cyclophilin A (PPIA) *C*_t_ variations in PNT2, LNCaP, MCF-7 and HEK cell lines according to hypoxic culture conditions (1, 5, 10 and 20% oxygen). cDNA (500 ng) –provided by reverse transcription of 500 ng of total RNA – was systematically used for qRT-PCR.

As an example of HKG variation, PPIA *C*_t_ fluctuations are represented (Figure 1). Results show that PPIA is not expressed at the same basal level in the different cell lines and varies regarding hypoxic conditions. Furthermore, it appears clearly that PPIA variations strongly differ according to cell lines, suggesting that PPIA is regulated in a cell-line-specific way. These variations of one of the most commonly used HKGs prompted us to verify whether other HKGs show same kind of variations.

**Figure 2:**
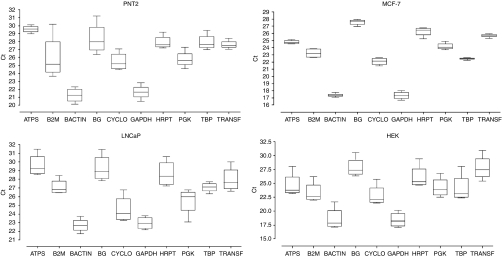
Housekeeping genes variations in PNT2, MCF-7, LNCaP and HEK cell lines grown in different hypoxic conditions (1, 5, 10 and 20% oxygen). Box plots represent *C*_t_ variations measured for each gene.

A global graph (Figure 2) shows all tested HKGs in each cell line. As for PPIA, other HKGs fluctuate according to hypoxic conditions and cell lines. A Friedman test based on means of HKG expression for each oxygen concentration indicates that HKG variation is statistically significant between different oxygen concentrations in PNT2 (*P*=0.0006), LNCaP (*P*<0.0001) and HEK (*P*<0.0001) cell lines. However, the difference in MCF-7 cell line remains insignificant (*P*=0.356), as shown in Figure 2 where box plots are largely thinner than those in other cell lines.

### Statistical analysis of hypoxia-based HKG variation

First, *C*_t_ coefficients of variations (CtCV%) were calculated for each HKG in each cell line (Table 2). As suggested by Figure 2, each HKG strongly varies regarding cell lines, as it is pinpointed for the *ACTB* gene whose CtCV% ranges from 1.41% in MCF-7 cell line to 9.77% in HEK cell line. Moreover, the best HKG differs from a cell line to another.

Second, the expression stability of the different putative HKG was tested using mathematical models. Two are yet available thus data obtained for each sample and each HKG were analysed using both GeNorm and NormFinder (Vandesompele *et al*, 2002; Andersen *et al*, 2004). GeNorm provides a ranking of the tested genes based on the reference gene stability measure *M*, which is defined as the average pair-wise variation of a particular gene compared with all other control genes. Genes with higher *M* values have greater variations of expression (Vandesompele *et al*, 2002). NormFinder, whose strategy is rooted in a mathematical model of gene expression, provides a ranking of the tested genes based on a direct measure of both overall expression variation and the variation between sample subgroups of candidate reference genes. The stability of genes was shown as stability value, and genes with lower stability values have higher expression stability (Andersen *et al*, 2004).

Results of the different calculations are summarised in Table 3, where all HKGs are classified for each cell line and for each statistical model according to their rank. It appears clearly that HKGs varying the least differ according to the cell line considered, whatever the statistical approach used. Furthermore, as it is exposed with highlighted *ATP5G3* in Table 3, GeNorm, NormFinder and CtCV% calculation do not give identical ranking of results. For example, in PNT2 cell line, when statistical analysis is performed either on CtCV% or NormFinder, *ATP5G3* seems to be the best HKG for normalisation but when GeNorm is used, ATP5G3 is likely to be one of the worst. In LNCaP cells, *GAPDH* appears the best HKG for GeNorm and NormFinder, the second best using CtCV% but it is truly the worst for the three methods in MCF-7 cells. In addition, when HEK cells are analysed, whereas *GAPDH* seems a relevant HKG on the basis of CtCV%, GeNorm and NormFinder considered *GAPDH* as the worst HKG.

Altogether, these results confirm that HKGs used for normalising gene expression in qRT-PCR may vary. Moreover, they emphasise that HKGs may dramatically fluctuate according to hypoxic conditions.

### Gene expression varies according to the HKG used

**Figure 3:**
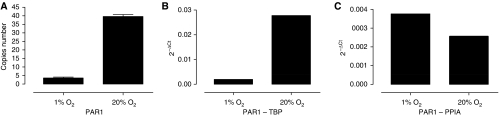
Gene expression varies according to the HKG used. In LNCaP cell line treated in two oxygen concentrations (1 and 20%), proteinase activating receptor gene 1 (*PAR1*) was (**A**) quantified alone with a standard curve, (**B**) normalised with the most stable HKG TBP or (**C**) normalised with one of the weakest stable HKG cyclophilin A (PPIA).

To evaluate the impact of HKG choice may have on the study of a target gene of interest, we performed qRT-PCR on RNAs extracted from LNCaP cells cultured in 1 and 20% oxygen. From the two derived cDNAs, the target gene, encoding PAR1 receptor known to be expressed and regulated in prostate carcinoma and cell lines (Kaushal *et al*, 2006; Yuan and Lin, 2004), was quantified, as well as two dedicated HKGs, namely TBP and PPIA. Both were chosen for their discrepant expression under variable hypoxic conditions: TBP showed one of the greatest stability in LNCaP cell line whereas PPIA was one of the less stable HKG in this model system. To quantify PAR1 transcript expression, we performed an absolute quantification. For that purpose, a standard curve was established using serial dilutions of a recombinant plasmid containing verified full-length PAR1 clone. This plasmid was used to determine the exactitude of the technique (intra (*n*=30) and inter (*n*=15) CtCV% <3.5 and 4.2% respectively), and to calculate the number of PAR1 equivalent copies present in test samples. When HKG normalisation is used, the observed results (Figure 3) unambiguously show that PAR1 is upregulated in normoxia when TBP is the reference HKG (Figure 3B), whereas it appears strongly downexpressed when PPIA is used for calculations (Figure 3C). In addition, both PAR1 quantification by standard curve (Figure 3A) and PAR1 normalisation by TBP show the same profile, suggesting that using HKG is not necessarily the best method to study the variation of a specific target gene. To validate PAR1 downregulation in hypoxia, we performed immunohistochemistry experiments that confirm at the protein level the decrease of PAR1 onto the plasma membrane of hypoxic LNCaP cells (data not shown).

## Discussion

The development of qRT-PCR over the past decade allowed a rapid and easy way to study gene expression. All qRT-PCR assays are characterised by biological variation, mainly the inherent variability of mRNA levels in different tissues and individuals, but also analytical variations characterised by differences in RNA quality, variability in extraction protocols, RT and PCR efficiencies that can lead to erroneous and confusing results (Huggett *et al*, 2005). One of the main drawbacks of qRT-PCR technique remains the difficulty to normalise results conveniently. Several normalisation methods have been proposed but none has gained universal approval (Huggett *et al*, 2005). Normalisation can be performed by different means: (1) conventional normalisation may use accurate measurement then normalisation against total RNA concentration, and can produce biologically relevant quantitative results (Tricarico *et al*, 2002). However, this method does not provide internal control for either RT or PCR. In addition, before any RT–PCR measurement, it requires a very accurate quantification of the RNA sample that is difficult to achieve (Bustin *et al*, 2005). A derived method can use rRNA normalisation (Bhatia *et al*, 1994; Zhong and Simons, 1999), which may result in less variability (Bas *et al*, 2004), nevertheless some fluctuations in rRNA normalisation have also been reported (Spanakis and Brouty-Boye, 1994); (2) the widely used method consists in using one or several HKGs, whose expression is assumed to be constant whatever the experimental or physiological conditions. Hence, using such normalised gene expression allows correcting data for cellular variability, RT efficiency and RNA quality. It is now accepted that normalisation against a single reference mRNA is likely to be inappropriate (Tricarico *et al*, 2002; Andersen *et al*, 2004), except in very well-defined circumstances (Dheda *et al*, 2004), but normalisation against a panel of reference genes, containing at least the three less variable, should be preferred (Vandesompele *et al*, 2002). Unfortunately, even using a panel of HKG, many studies have pinpointed that HKG can fluctuate in cell lines as well as in tissue (Thellin *et al*, 1999; Schmittgen and Zakrajsek, 2000; Lupberger *et al*, 2002; Schmid *et al*, 2003; Aerts *et al*, 2004; Biederman *et al*, 2004; Dheda *et al*, 2004; de Kok *et al*, 2005; Synnergren *et al*, 2007; Cicinnati *et al*, 2008). Among the more recent ones, Derks *et al* (2008) showed that HKG expression was not constant enough to be used as internal control for gene normalisation in rat brains. The same conclusions were raised for melanoma and fibroblasts samples (Giricz *et al*, 2008).

The choice of a relevant HKG has to rely on observed unambiguous data regarding the experimental system where it is supposed to be used. Unfortunately, for most of the HKG used in published results, no exhaustive studies of their variations in dedicated conditions have been performed yet. For example, a lot of reports deal with transcripts variation under hypoxia (Helczynska *et al*, 2008; Seifeddine *et al*, 2008; Sheikh *et al*, 2008; Chaudary and Hill, 2009; Zhang *et al*, 2009). Authors formulate relevant conclusions regarding gene expression on the basis of the use in most cases of a unique or more rarely a specific set of HKG. However, the variations of that unique gene or set of genes have not been much studied, especially in the case of hypoxic conditions. If, as it was shown for some instances of HKG in very controlled conditions, HKG vary also under hypoxia, then a large number of results analysing transcript regulation under hypoxia have to be interpreted with the utmost care. Thus, as potential users of HKG to evaluate gene expression under hypoxia, we aimed to evaluate to which extent widely used HKG in qRT-PCR may vary in cell lines cultured in different oxygen concentrations. Our study results unambiguously show that HKG expression dramatically fluctuates depending on air oxygen content. Whatever the HKG measured, slight to important variations in expression can be observed in an oxygen-concentration-dependent manner. Moreover, although these variations differ from one HKG to another, we observed surprisingly that variation of a dedicated HKG may differ from one cell line to another. Indeed, HKG harbouring a good CtCV% in a given cell line may show a higher coefficient of variation in another cell line (Table 2). This holds particularly true for *GAPDH*, which oscillates with the second larger amplitude after B2M according to hypoxia conditions and cell lines studied. This confirms previous results that have already been reported *GAPDH* variations in various prostate and endothelial cell lines (Zhong and Simons, 1999). Nevertheless, among the cell lines we tested, some seem to be more or less responsive to hypoxic treatments than others, as it is suggested for MCF-7 cell line where HKG variations appears to be not significant. Our study results corroborate the conclusions raised by Haller *et al* (2004) on HKG variations in several carcinomas who stated that a stable expression of a reference gene in one tumour type does not predict a stable expression in another.

Indeed, top choice of HKG is challenging because of the impact it could have on the results reliability and, as a consequence, on the results interpretations. Indeed, using HKG whose expression will vary in opposite directions under the same stimulus will undoubtedly led to errors in interpretation as it was shown above on PAR1 transcripts that are upregulated or downregulated by hypoxia according to the HKG used for the experiment (Figure 3). Here, we found by both absolute quantification and best HKG normalisation (using TBP in LNCaP hypoxic system) that PAR1 transcripts are likely to be downregulated in that prostate cancer cell line. This was further validated at the protein level confirming PAR1 global downregulation in LNCaP cells treated in hypoxic conditions. However, a recent report showed that, in MDAMDB321 aggressive breast cancer cells, hypoxia (2% oxygen) enhance PAR1 expression on the basis of conventional qRT-PCR experiments using ACTB as HKG, gel analysis for transcript quantification (Naldini *et al*, 2009), but no reported data regarding the choice of ACTB. Nevertheless, in our hands, MCF-7 breast cancer cells appear to have the most stable HKGs in hypoxia. Among them, *ACTB* displays a middle range CtCV% (1.41%) and is relatively stable. However *MCF-7* could not be superimposed to MDAMDB321 cells as great expression variability can be observed between cell lines of the same tissue or embryonic origin as shown by prostate PNT2 and LNCaP cells. Thus, no clear conclusions concerning the rationale of the use of ACTB can be extrapolated to MDAMB321 cells system. Hence, PAR1 regulation appears strongly modulated by hypoxia during natural course of cancer, emphasising the need of a very careful quantification of its variation at either transcript or protein levels. For a unique specific target or a small set of specific targets, confirmation of transcript variation by further evaluation of protein variation by either immunohistochemistry or western blot will undoubtedly validate the initial choice of the best HKG.

Nevertheless, for large-scale studies of gene expression (transcripts variation analysis by microarrays, microfluidic qRT-PCR and so on) for which validation at the protein level is quite impossible, a clear validation step of suitable reference genes remains a crucial problem. Different statistical approaches (GeNorm, NormFinder) have been developed to identify and validate reference genes appropriate for normalisation in qRT-PCR assays (Andersen *et al*, 2004; Haller *et al*, 2004; Vandesompele *et al*, 2002). However, the use of these devoted software does not elucidate the problem of the best HKG choice, as shown in Table 3. Indeed, both statistical approaches do not validate the same genes according to a given cell line. Moreover, as it was observed in CtCV% calculation, these approaches pinpoint HKG undulations according to cell lines and hypoxia conditions.

Taken together, the results of our study suggest that neither a single gold standard HKG nor a set of HKG can be widely used for qRT-PCR normalisation in hypoxia experiments. Therefore, it seems essential to test HKG expression before any experiment based on hypoxic culture. Besides for each cell line, the best HKG should also be specifically determined and validated before conducting hypoxic studies. An additional and fundamental question that remains to be determined before any analysis concerns the choice of the most convenient statistical approach that should be used to identify the best HKG or set of HKG. Whereas GeNorm and NormFinder have been reported to be interesting statistical means to choose the best HKG in specific normal or tumour tissues (Cicinnati *et al*, 2008; Lyng *et al*, 2008), a careful evaluation of their use in different *in vitro* experiments with a multiplicity of experimental conditions is necessary.

However, in the case of a unique target gene of interest, in very well-defined *in vitro* conditions where it is possible to have equivalent amount of starting material, the use of a standardised curve for absolute quantification is likely to be the best way to study its variations. Otherwise, in all other cases where multiple set of target genes in various conditions have to be analysed, each set of HKG must be tested before any normalisation in the cell lines and under the conditions the assay will be performed. Till universal validation of relevant statistical software, results have to be carefully analysed regarding the CtCV% of each HKG. Once the more stable HKG or set of HKG in that condition has been evidenced as the best for normalisation, then it will be really possible to begin qRT-PCR experiments.

The choice of a valid HKG set will undoubtedly determine the relevance of the results that will be further interpreted, and so it should be seriously considered. Indeed, such kind of assay design, functional for all *in vitro* systems, should be implemented before any extension to other experimental models including *in vivo* ones. Nevertheless, one should keep in mind that defining a universal and unanimous method to choose the best HKG in a system model is still strongly mandatory.

## Figures and Tables

**Table 1:** Characteristics of the different selected housekeeping genes

**Gene name**	**Abbreviation**	**Function**	**Accession IDs**	**5′-Primers-3′ (forward/reverse)**	**Size (bp)**
ATP synthase, H+ transporting, mitochondrial F0 complex, subunit C3 (subunit 9)	*ATP5G3*	Oxydative phosphorylation	NM_001002258.4	GGATTTGCCTTGTCTGAAGC CGTACATTCCCATGACACCA	188
*β*-2-Microglobulin	*B2M*	*β*-Chain of major histocompatibility complex class 1 molecules	NM_004048	CTCACGTCATCCAGCAGAGA TCTTTTTCAGTGGGGGTGAA	198
*β*-Actin	*ACTB*	Cytoskeletal structural protein	NM_001101	GGGGTGTTGAAGGTCTCAAA GGCATCCTCACCCTGAAGTA	183
*β*-Glucuronidase	*GUSB*	Exoglycosidase in lysosomes	NM_000181	CTGTACACGACACCCACCAC ATTCGCCACGACTTTGTT	208
Cyclophilin A	*PPIA*	Serine–threonine phosphatase inhibitor	NM_021130	ACCGTGTTCTTCGACATTGC GGCATGAATATTGTGGAGGC	410
Glyceraldehyde-3-phosphate dehydrogenase	*GAPDH*	Glycolysis enzyme	NM_002046	GAGTCCACTGGCGTCTTCAC GGTGCTAAGCAGTTGGTGGT	177
Hypoxanthine phosphoribosyltransferase 1	*HPRT1*	Hydrolase in carbohydrate metabolism	NM_000194	TGCTCGAGATGTGATGAAGG TCCCCTGTTGACTGGTCATT	181
Phosphoglycerate kinase 1	*PGK1*	Glycolysis enzyme	L00159	GAAGTGGAGAAAGCCTGTGC CTCTGTGAGCAGTGCCAAAA	159
TATA-box-binding protein	*TBP*	General RNA polymerase II transcription factor	M55654	TGCCTCCAGAATATGCCTCT CAATGGTTTTCAAGCTTTCCA	203
Transferrin receptor (p90, CD71)	*TFRC*	Cellular iron uptake	X01060	GGAGAATCCTGGGGGTTATG GCTTTCAGCATTTGCAACCT	202

For each gene, both forward and reverse primers sequences are given with the expected PCR amplimer size.

**Table 2:** Coefficients of variation (CtCV%) of housekeeping genes

	**PNT2**	**LNCaP**	**MCF-7**	**HEK**	**All**
*ATP5G3*	1.66	3.96	1.02	8.04	9.96
*B2M*	11.16	2.98	2.5	7.29	9.33
*ACTB*	4.53	3.15	1.41	9.77	11.83
*GUSB*	7.35	4.8	1.32	5.82	5.51
*PPIA*	4.66	5.75	2.07	7.61	7.75
*GAPDH*	4.51	2.87	2.83	6.64	12.23
*HRPT1*	3.18	4.75	2.31	7.41	6.15
*PGK1*	4.34	5.58	1.83	6.78	5.63
*TBP*	3.71	1.89	0.58	9.54	10.02
*TFRC*	2.06	4.64	0.95	7.25	5.65

**Table 3:** HKG variations summary in PNT2, LNCaP, MCF-7 and HEK cell lines grown in different oxygen concentrations

	**PNT2**			**LNCaP**			**MCF-7**			**HEK**		
**Rank**	**CtCV%**	**NormFinder**	**GeNorm**	**CtCV%**	**NormFinder**	**GeNorm**	**CtCV%**	**NormFinder**	**GeNorm**	**CtCV%**	**NormFinder**	**GeNorm**
1	*ATP5G3*	*ATP5G3*	*HPRT1*	*TBP*	*GAPDH*	*GAPDH*	*TBP*	*ATP5G3*	*TBP*	*GUSB*	*PPIA*	*PPIA*
2	*TFRC*	*B2M*	*TBP*	*GADPH*	*ATP5G3*	*ATP5G3*	*TFRC*	*TBP*	*ATP5G3*	*GAPDH*	*HPRT1*	*HPRT1*
3	*HPRT1*	*ACTB*	*PGK1*	*B2M*	*HPRT1*	*HPRT1*	*ATP5G3*	*TFRC*	*GUSB*	*PGK1*	*B2M*	*B2M*
4	*TBP*	*GUSB*	*PPIA*	*ACTB*	*TFRC*	*GUSB*	*GUSB*	*GUSB*	*TFRC*	*TFRC*	*PGK1*	*ATP5G3*
5	*PGK1*	*PPIA*	*TFRC*	*ATP5G3*	*GUSB*	*TFRC*	*ACTB*	*ACTB*	*ACTB*	*B2M*	*ATP5G3*	*PGK1*
6	*GAPDH*	*GAPDH*	*GAPDH*	*TFRC*	*TBP*	*TBP*	*PGK1*	*PGK1*	*PGK1*	*HPRT1*	*TFRC*	*GUSB*
7	*ACTB*	*HPRT1*	*ATP5G3*	*HPRT1*	*ACTB*	*ACTB*	*PPIA*	*PPIA*	*PPIA*	*PPIA*	*GUSB*	*TFRC*
8	*PPIA*	*PGK1*	*ACTB*	*GUSB*	*PPIA*	*PPIA*	*HPRT1*	*HPRT1*	*HPRT1*	*ATP5G3*	*TBP*	*TBP*
9	*GUSB*	*TBP*	*GUSB*	*PGK1*	*PGK1*	*PGK1*	*B2M*	*B2M*	*B2M*	*TBP*	*ACTB*	*ACTB*
10	*B2M*	*TFRC*	*B2M*	*PPIA*	*B2M*	*B2M*	*GAPDH*	*GAPDH*	*GAPDH*	*ACTB*	*GAPDH*	*GAPDH*

HKGs are ranked according to their stability calculated by three different statistical approaches: coefficient of variations (CtCV%) calculations, and NormFinder and GeNorm software.
